# Bile acid metabolites enhance expression of cathelicidin antimicrobial peptide in airway epithelium through activation of the TGR5-ERK1/2 pathway

**DOI:** 10.1038/s41598-024-57251-3

**Published:** 2024-03-21

**Authors:** Iwona T. Myszor, Kornelia Lapka, Kristjan Hermannsson, Rokeya Sultana Rekha, Peter Bergman, Gudmundur Hrafn Gudmundsson

**Affiliations:** 1 Faculty of Life and Environmental Sciences, Biomedical Center, University of Iceland, Reykjavik, Iceland; 2 Department of Laboratory Medicine, Karolinska Institutet, Stockholm, Sweden; 3 Department of Clinical Immunology and Transfusion Medicine, Karolinska University Hospital, Stockholm, Sweden

**Keywords:** Antimicrobial responses, Innate immunity

## Abstract

Signals for the maintenance of epithelial homeostasis are provided in part by commensal bacteria metabolites, that promote tissue homeostasis in the gut and remote organs as microbiota metabolites enter the bloodstream. In our study, we investigated the effects of bile acid metabolites, 3-oxolithocholic acid (3-oxoLCA), alloisolithocholic acid (AILCA) and isolithocholic acid (ILCA) produced from lithocholic acid (LCA) by microbiota, on the regulation of innate immune responses connected to the expression of host defense peptide cathelicidin in lung epithelial cells. The bile acid metabolites enhanced expression of cathelicidin at low concentrations in human bronchial epithelial cell line BCi-NS1.1 and primary bronchial/tracheal cells (HBEpC), indicating physiological relevance for modulation of innate immunity in airway epithelium by bile acid metabolites. Our study concentrated on deciphering signaling pathways regulating expression of human cathelicidin, revealing that LCA and 3-oxoLCA activate the surface G protein-coupled bile acid receptor 1 (TGR5, Takeda-G-protein-receptor-5)—extracellular signal-regulated kinase (ERK1/2) cascade, rather than the nuclear receptors, aryl hydrocarbon receptor, farnesoid X receptor and vitamin D3 receptor in bronchial epithelium. Overall, our study provides new insights into the modulation of innate immune responses by microbiota bile acid metabolites in the gut-lung axis, highlighting the differences in epithelial responses between different tissues.

## Introduction

Primary bile acids are steroid-like molecules that are important for the dietary lipid emulsification and metabolism^1^. They exhibit direct and indirect antimicrobial properties in the small intestine, where the indirect effects are achieved by induction of antimicrobial effectors through the stimulation of the nuclear receptor FXR (farnesoid X receptor; NR1H4)^2^. A large portion of primary bile acids is reabsorbed from the small intestine to the liver through the portal vein. During the process, a small fraction of primary bile acids can enter peripheral circulation reaching low concentrations in blood of approximately 2–10 µM^3^.

The remaining pool of primary bile acids is further metabolized by colonic microbiota to secondary bile acids and their metabolites that shape host innate and adaptive immune responses, maintaining local tissue homeostasis^1^. Lithocholic (LCA) and deoxycholic (DCA) acids were shown to reduce inflammation in a mouse model of colitis through the stimulation of the surface G protein-coupled bile acid receptor 1 (GPBAR1 or TGR5) in immune cells^4^. Also, two microbiota metabolites of LCA, 3-oxolithocholic acid (3-oxoLCA) and alloisolithocholic acid (AILCA), were shown to affect regulation of adaptive immune responses connected to the differentiation of T cells into specific cell subsets in the gut lamina propria, providing local tissue homeostasis^5–7^. Proliferation of proinflammatory Th17 cells was inhibited by 3-oxoLCA, while AILCA stimulated differentiation of anti-inflammatory Treg cells^5^.

Interestingly, microbiota bile acid metabolites have been detected in blood, their levels vary between individuals and can fluctuate for example, upon antibiotic intake^8^. Moreover, bile acid metabolites circulating in blood can affect gastrointestinal (GI)-distant tissues. Deoxycholic acid was shown to alter production and differentiation of bone marrow granulocyte-monocyte progenitors providing protection from infection with *Entamoeba histolytica*^9^. This indicates that bile acid metabolites produced by colonic microbiota by entering the blood circulation can shape epithelial innate immune responses in GI-distant tissues, including those connected to the expression of host defense peptides (HDPs). Cathelicidin is one of the important HDPs, the only member of the cathelicidin family in humans, encoded by the *CAMP* gene and produced as a cathelicidin proform (pro-LL-37) that upon processing releases mature LL-37 peptide with antimicrobial and immunomodulatory properties. Together with several components of innate immunity, cathelicidin provides the front-line defenses on the mucosal surfaces^10–13^.

In our study, we analyzed whether bile acid metabolites induce production of HDPs in airway epithelial cells to enhance host front-line defenses and what is the molecular mechanism of cathelicidin induction. Due to a high redundancy of bile acid metabolites and their receptors, we sought to determine which receptors in airway epithelium are targets for bile acid metabolites and identify signaling pathways involved in the induction of cathelicidin expression. Our study provides a new insight into the characterization of microbiota metabolites as signaling molecules affecting host innate immune responses in GI-distant mucosal surfaces.

## Results

### Screening of lithocholic acid derivatives for cathelicidin induction

**Figure 1:**
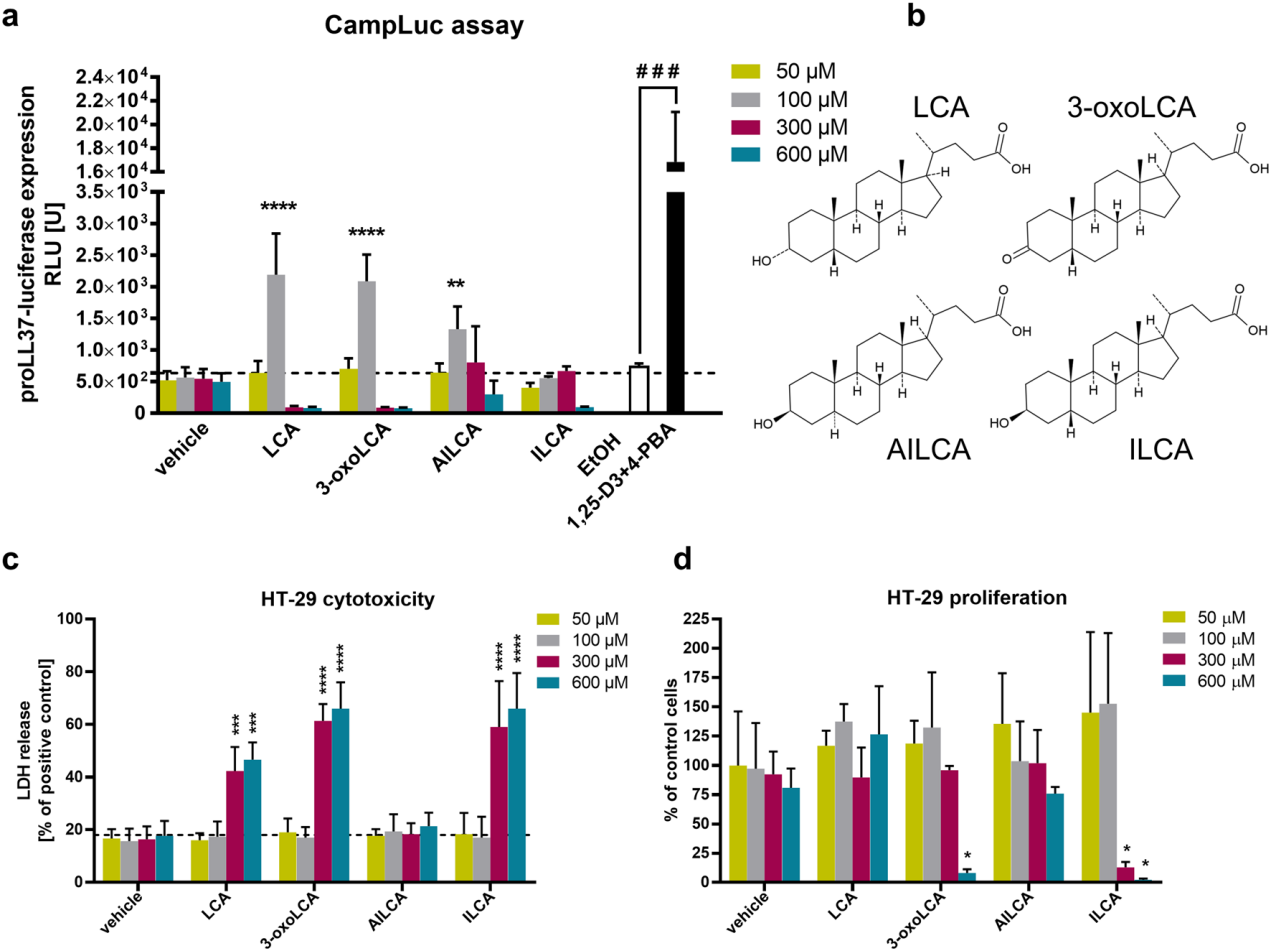
Microbiota bile acid metabolites induce expression of the proLL37-luciferase fusion protein. (**a**) The CampLuc assay with HT-29 cell line expressing the proLL37-luciferase fusion protein upon induction with different concentrations (50–600 µM) of lithocholic acid (LCA) and LCA derivatives: 3-oxolithocholic acid (3-oxoLCA), alloisolithocholic acid (AILCA), isolithocholic acid (ILCA) which chemical structures are presented in (**b**). The treatment with 1,25-dihydroxyvitamin D3 (1,25-D3; 100 nM) and 4-phenylbutyrate (4-PBA; 2 mM) was used as a positive control for the induction of the fusion protein and ethanol (EtOH; 0.5% v/v) was used as a solvent control for 1,25-D3. (**c**) Cytotoxic effect of LCA and derivatives (50–600 µM) on parental HT-29 cell line measured as a release of LDH (lactate dehydrogenase) and calculated as a percentage of the positive control (100%). (**d**) Effect of LCA and derivatives (50–600 µM) on the proliferation of parental HT-29 cell line measured as a percentage of the proliferation of control (untreated) cells. Experiments were performed at 24 h post induction, n = 3 independent experiments, each performed in triplicates ± SD. Statistical analysis was performed by two-way Anova with Dunnett’s multiple comparisons post-hoc test where, for the *p* values *< 0.05, **< 0.01, ***< 0.001 and ****< 0.0001. One-way Anova with Sidak’s multiple comparisons post-hoc test was used to compare the treatment with 1,25-D3 and 4-PBA to corresponding vehicle (ethanol) control, where ^###^< 0.001.

To test whether different bile acid metabolites can induce expression of HDPs in epithelial cells, we first applied a luciferase reporter established in HT-29 cells (CampLuc cells) that was developed for screening for potential inducers of cathelicidin expression^14^. In the CampLuc assay, we tested if the following bile acid metabolites: lithocholic acid (LCA), 3-oxolithocholic acid (3-oxoLCA), alloisolithocholic acid (AILCA), isolithocholic acid (ILCA) could induce expression of proLL37-luciferase fusion protein (Fig. 1a,b). The treatment with 4-phenylbutyrate (4-PBA) and 1,25-dihydroxyvitamin D3 (1,25-D3) served as a positive control for induction of the cathelicidin expression as demonstrated previously^15^. LCA was included for the comparison because it was shown previously to induce cathelicidin expression in parental HT-29 cells^16^. In addition, LCA serves as a substrate for the synthesis of 3-oxoLCA, AILCA and ILCA by commensal bacteria in human colon^6^. Using different concentrations of bile acid metabolites at 50–600 µM, we showed that LCA, 3-oxoLCA and AILCA induced expression of the proLL-37-luciferase fusion protein at 100 µM in HT-29 luciferase reporter cell line (Fig. 1a). The concentration of 100 µM for LCA, 3-oxoLCA and AILCA did not exhibit any cytotoxic effect on the parental HT-29 cell line, which was observed when higher concentrations of bile acid metabolites were used at 300 and 600 µM (Fig. 1c). Moreover, the selected concentration of 100 µM for LCA, 3-oxoLCA and AILCA did not inhibit proliferation of parental HT-29 colonic epithelial cells (Fig. 1d). Together, these results demonstrate that selected bile acid metabolites at the concentration of 100 µM induce cathelicidin expression in colonic epithelial cells without displaying cytotoxic effects.

### Induction of cathelicidin by lithocholic acid derivatives in the airway epithelium

**Figure 2:**
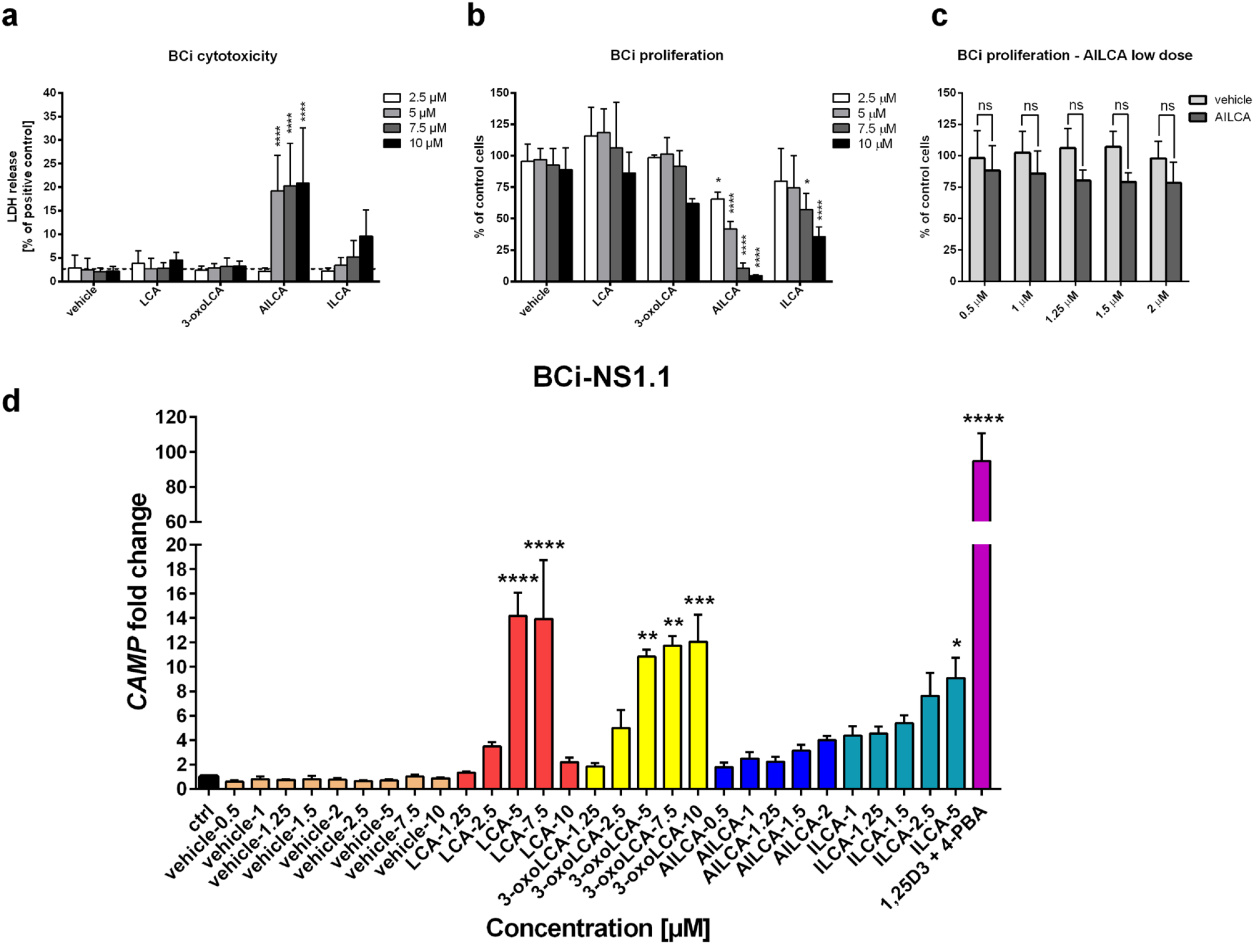
Microbiota bile acid metabolites induce expression of cathelicidin in human bronchial epithelial BCi cells at low doses. (**a**) Cytotoxic effect of LCA and derivatives: 3-oxoLCA, AILCA and ILCA (2.5–10 µM) on BCi cells measured as a release of LDH (lactate dehydrogenase) and calculated as a percentage of positive control (100%). (**b**) Effect of LCA and derivatives on the proliferation of BCi cells at concentration range 2.5–10 µM measured as a percentage of control (untreated) cells proliferation. (**c**) Effect of alloisolithocholic acid (AILCA) on BCi proliferation at low concentrations of up to 2 µM measured as a percentage of control (untreated) cells proliferation. (**d**) Expression of cathelicidin (*CAMP*) gene in bronchial epithelial cells by LCA and 3-oxoLCA (2.5–10 µM), AILCA (0.5–2 µM) and ILCA (1–5 µM). Treatment with combination of 1,25-dihydroxyvitamin D3 (1,25D3; 100 nM) and 4-phenylbutyrate (4-PBA; 2 mM) was used as a positive control for induction of the *CAMP* expression. Expression of *CAMP* gene was analyzed by qRT-PCR using *EEF2* housekeeping gene and is presented as *CAMP* fold change over control (untreated cells) that has value 1. All experiments were performed 24 h post treatment, n = 4 ± SD for (**a–c**) and n = 3 ± SD for (**d**). Statistical analysis was performed with two-way Anova with Dunnett’s and Sidak’s post-hoc tests for (**a–b**) and (**c**), respectively. One-way Anova with Sidak’s post-hoc test was used to compare the treatment with microbiota bile acid metabolites at specific concentration to the corresponding vehicle control in (**d**). For all graphs the *p* values are *< 0.05, **< 0.01, ***< 0.001, ****< 0.0001 and *ns* non significant.

After confirming induction of cathelicidin by bile acid metabolites in the CampLuc assay, we investigated whether bile acid metabolites could modulate expression of HDP cathelicidin in different types of epithelial cells, other than colonic epithelium. Therefore, we used the human bronchial epithelial cell line BCi with stem cell-like character and human primary bronchial/tracheal epithelial cells (HBEpC) from healthy donors to determine the non-cytotoxic concentration range for bile acid metabolites in the lung model in vitro. Among four bile acid metabolites used within 2.5–10 µM concentration range, LCA, 3-oxoLCA and ILCA did not have any cytotoxic effect on BCi cells (Fig. 2a). On the other hand, AILCA showed the highest cytotoxicity (Fig. 2a), that inhibited BCi proliferation at low concentrations (Fig. 2b). ILCA inhibited BCi proliferation at 7.5 and 10 µM, and AILCA required an additional titration experiment within 0.5–2 µM range to find the dose that did not inhibit cell proliferation (Fig. 2c). After initial characterization of bile acid metabolites cytotoxicity and inhibitory concentration for airway epithelial cells, we aimed to determine whether, selected non-cytotoxic doses of bile acid metabolites could induce cathelicidin expression at RNA level in BCi cells (Fig. 2d). Among bile acid metabolites, LCA and 3-oxoLCA were tested at concentrations reaching up to 10 µM, that induced cathelicidin (*CAMP*) gene expression more than tenfold at 5 and 7.5 µM. Further, ILCA induced *CAMP* expression only at the highest concentration of 5 µM that did not inhibit BCi proliferation, while AILCA did not have any significant effect on *CAMP* induction (Fig. 2d). These results indicate that bile acid metabolites LCA, 3-oxoLCA and ILCA induce expression of cathelicidin in human bronchial epithelial cells at low concentrations.

### Induction of the cathelicidin proform in BCi and human primary cells by bile acid metabolites

**Figure 3:**
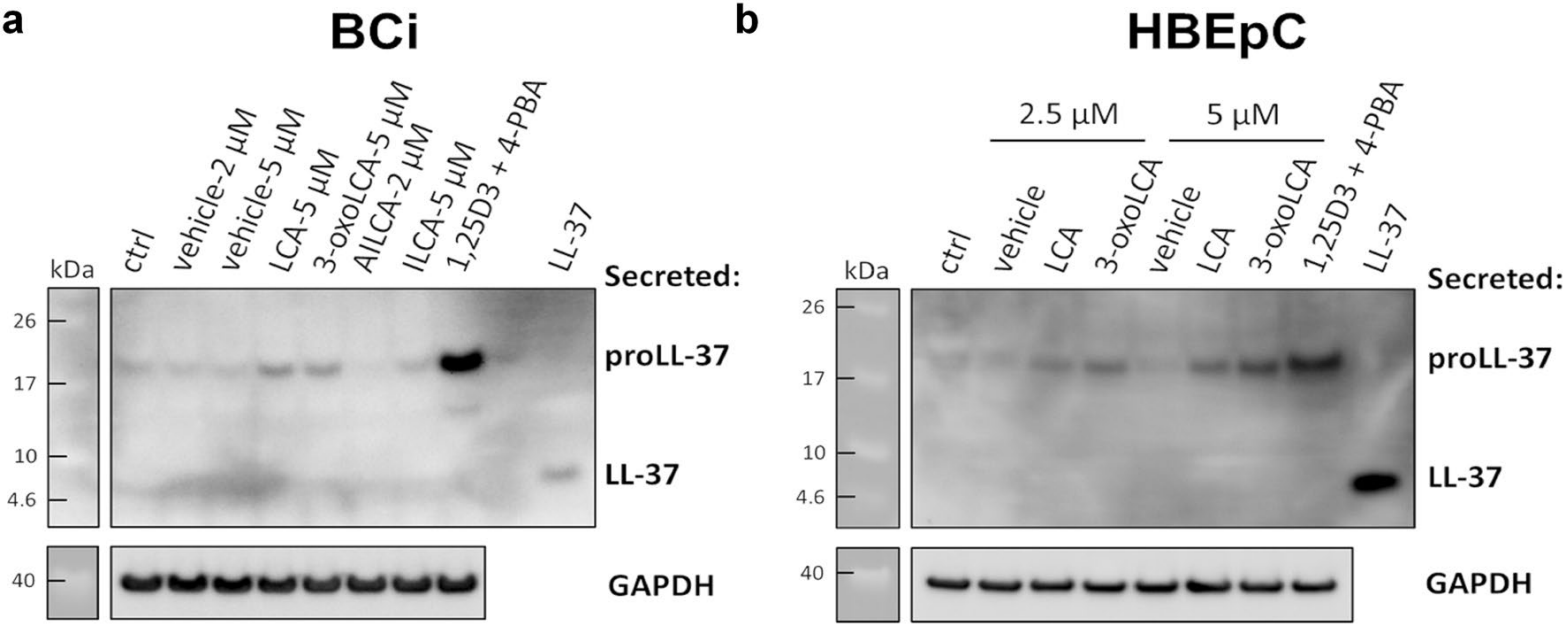
Lithocholic and 3-oxolithocholic acids induce expression of the cathelicidin proform. Expression of the cathelicidin proform (proLL-37) and processing to the mature LL-37 peptide was analyzed in protein enriched cell culture conditioned media after 24 h post treatment of (**a**) BCi-NS1.1 cells (BCi) and (**b**) human bronchial/tracheal epithelial primary cells (HBEpC) with LCA and derivatives by Western blot. Treatment with 1,25-dihydroxyvitamin D3 (1,25D3; 100 nM) and 4-phenylbutyrate (4-PBA; 2 mM) was used as a positive control for induction of the proLL-37 expression. Synthetic human LL-37 peptide (sLL-37) was used as a standard control for Western blot analysis. Expression of GAPDH was analyzed in corresponding cell lysates and used as a loading control. n = 3 independent experiments; for HBEpC two independent experiments with cells from donor #2644 (passage 1 and 2) and one independent repeat with cells from donor #1865 (passage 2). Protein enriched cell culture conditioned media and corresponding cell lysates were run on separate gels processed in parallel and original blots are presented in Supplementary Fig. 1.

Bile acid metabolites induced cathelicidin gene expression in human bronchial epithelium therefore, in the next step we verified whether these changes translate into increased protein levels of cathelicidin in BCi cell line and human primary bronchial/tracheal epithelial cells (HBEpC) (Fig. 3). The concentration of 5 µM was selected for LCA, 3-oxoLCA and ILCA based on induction of the *CAMP* gene expression in BCi cells. In addition, we included the highest non-cytotoxic concentration of 2 µM for AILCA to exclude possible discrepancies between induction of cathelicidin at the RNA and protein level. In both, BCi and HBEpC bronchial epithelial cells, the processing of the cathelicidin proform (≈ 18 kDa) to mature LL-37 peptide was not observed and only the cathelicidin proform was detected (Fig. 3). Among tested bile acid metabolites, LCA and 3-oxoLCA induced expression of the pro-LL-37 in BCi cells, while no changes were observed for AILCA and ILCA (Fig. 3a). Based on that, we selected LCA and 3-oxoLCA for further study with HBEpC and tested both bile acid metabolites at 2.5 µM and 5 µM. Induction of cathelicidin expression by LCA and 3-oxoLCA was confirmed in human HBEpC (Fig. 3b). The lower concentrations of 2.5 µM induced the expression of the cathelicidin proform at similar levels as 5 µM in BCi cells. Overall, LCA and 3-oxoLCA induce expression of the cathelicidin proform in human bronchial epithelial cells.

### Nuclear receptors are not the major contributors to the bile acid-mediated induction of cathelicidin expression in the airway epithelium

**Figure 4:**
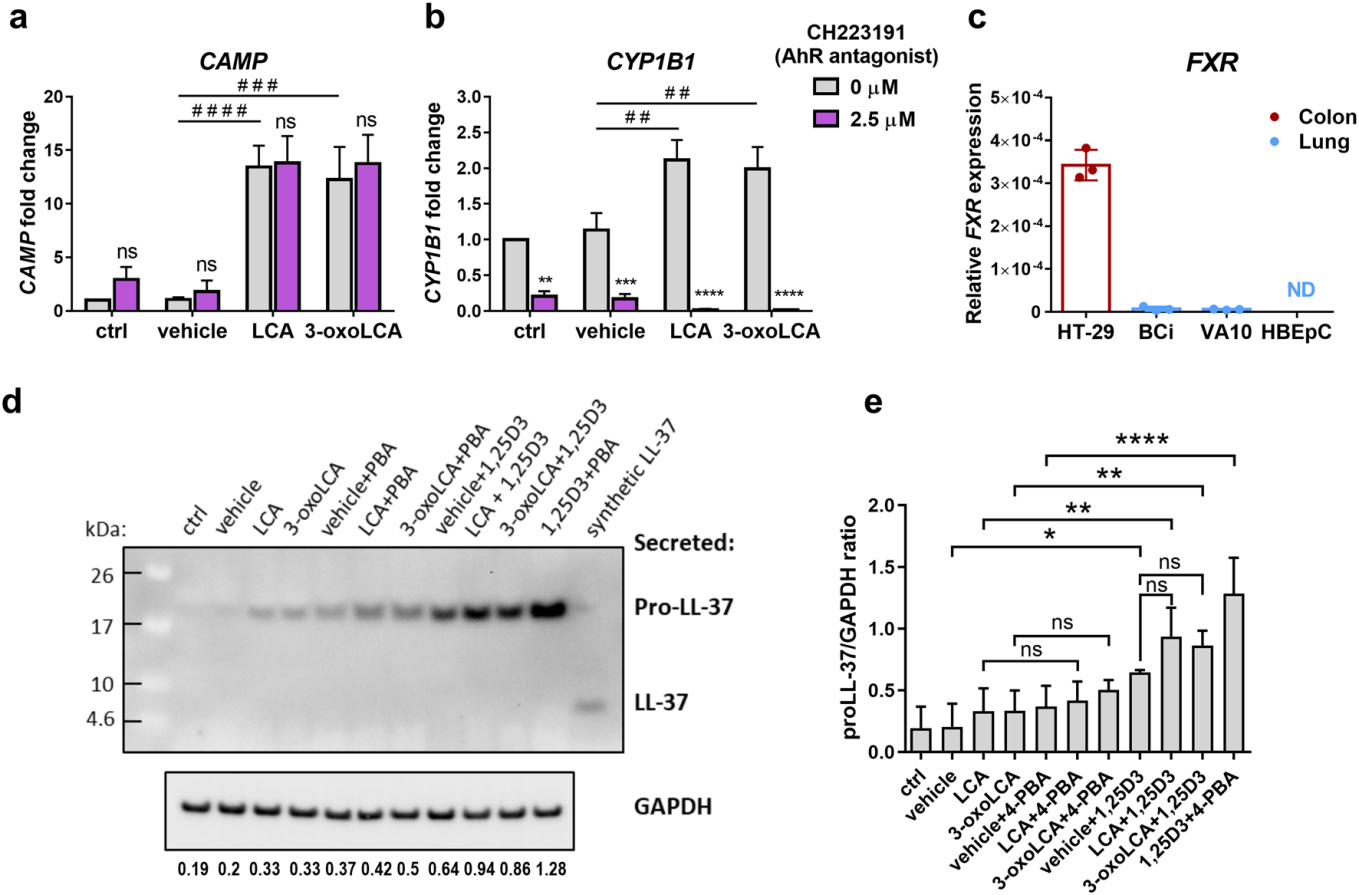
Induction of cathelicidin expression by bile acid metabolites is not mediated through selected nuclear receptors in bronchial epithelium. Expression of (**a**) cathelicidin (*CAMP*) and (**b**) cytochrome P450 family 1 subfamily B member 1 (*CYP1B1*) genes in BCi cells pretreated for 1 h with CH223191 (2.5 µM) followed by addition of lithocholic acid (LCA; 5 µM) and 3-oxolithocholic acid (3-oxoLCA; 5 µM) in the presence of CH223191 for the next 24 h analyzed by qRT-PCR and presented as a fold change over control (ctrl). n = 3 independent experiments ± SD analyzed by two- and one-way Anova with Sidak’s post-hoc test. The *p* values are **< 0.01, ***< 0.001, ****< 0.0001 and *ns* nonsignificant for two-way Anova used to compare CH223191-pretreated cells vs untreated cells within each bile acid treatment group and ^##^< 0.01, ^###^< 0.001, ^####^< 0.0001 for one-way Anova used to compare bile acid treatment to the vehicle control within the CH223191-untreated group. (**c**) Expression of farnesoid X receptor (*FXR*; *NR1H4*) in colonic HT-29 cell line, bronchial BCi and VA10 cell lines, and human primary bronchial/tracheal epithelial cells (HBEpC) presented as a relative expression 2^−ΔCt^ where *ND* not detected transcript, n = 3 independent experiments ± SD. (**d**) Expression of the cathelicidin proform (proLL-37) and processing to the LL-37 peptide was analyzed in cell conditioned media after 24 h post treatment of BCi cells by Western blot. Treatment with LCA and 3-oxoLCA was performed with and without 1,25-dihydroxyvitamin D3 (1,25D3; 100 nM) and 4-phenylbutyrate (4-PBA; 2 mM). A combination of 1,25D3 and 4-PBA was used as a positive control for induction of the proLL-37 and the synthetic human LL-37 peptide (sLL-37) was used as a standard control. Expression of GAPDH used as a loading control was analyzed in corresponding cell lysates, n = 3 independent experiments. Protein enriched cell culture conditioned media and corresponding cell lysates were run on separate gels processed in parallel and original blots are presented in Supplementary Fig. 1. (**e**) Quantification of the band intensity presented as a ratio of ProLL-37 to GAPDH from n = 3 independent experiments ± SD analyzed by one-way Anova with Sidak’s post-hoc test, where *p* values *< 0.05, **< 0.01, ****< 0.0001 and *ns* nonsignificant.

To delineate the mechanism of cathelicidin induction by LCA and 3-oxoLCA in bronchial epithelial BCi cells, we first concentrated on the nuclear receptors shown to regulate gene expression in response to bile acids and their metabolites^17^. We investigated the role of nuclear receptor aryl hydrocarbon receptor (AhR), which is an environmental sensor for xenobiotics and tryptophan metabolites produced endogenously by the host and also by microbiota^18^. The preincubation of BCi cells with the AhR antagonist CH223191 did not abrogate the induction of *CAMP* gene expression by LCA and 3-oxoLCA (Fig. 4a). In contrast, the AhR antagonist counteracted induction of the AhR downstream target gene *CYP1B1* by LCA and 3-oxoLCA (Fig. 4b). This indicates that the AhR stimulated by LCA and 3-oxoLCA is not involved in the regulation of *CAMP* expression in bronchial epithelium.

Regarding the regulation of antimicrobial responses, two nuclear receptors FXR (NR1H4) and VDR (NR1I1) were shown to be involved in the regulation of *CAMP* expression by chenodeoxycholic acid (CDCA) and ursodeoxycholic acid (UDCA) in the biliary epithelium of the liver^19^. Moreover, VDR is a transcription factor regulating the expression of human cathelicidin in human bronchial epithelial cells^20–23^ and it has been shown to be activated by LCA and 3-oxoLCA^17,24^. Therefore, we determined whether FXR and VDR are involved in the enhancement of the cathelicidin expression in response to LCA and 3-oxoLCA in bronchial epithelium. In our study bronchial epithelial cells, such as BCi and VA10 cell lines, and human primary HBEpC cells expressed *FXR* transcript at low levels or did not express it at all, in contrast to HT-29 cell line derived from colonic epithelium (Fig. 4c). This suggests that the FXR is not the major target receptor for LCA and 3-oxoLCA in our lung model and cathelicidin induction is mediated through stimulation of a different receptor/-s. For that reason, we included the co-treatment of LCA and 3-oxoLCA with 1,25-dihydroxyvitamin D3 (1,25D3) to evaluate the involvement of VDR (Fig. 4d). Addition of 1,25D3 further increased expression of the cathelicidin proform induced by LCA and 3-oxoLCA and the quantification of the band intensity indicated on the additive effect (Fig. 4e). On the other hand, the addition of LCA and 3-oxoLCA to 1,25D3 did not show significant increase (Fig. 4e). We also included the co-treatment with 4-phenylbutyrate (4-PBA), a weak inhibitor of histone deacetylase enzyme, which did not have any enhancing effect (Fig. 4d). Overall, the expression of cathelicidin at the protein level was higher in the co-treatment with 1,25D3 than with 4-PBA (Fig. 4d). Stimulation of VDR by 1,25D3 in the co-treatment with LCA and 3-oxoLCA exerted an additive induction of *CAMP* expression, that does not exclude involvement of additional pathways.

### LCA and 3-oxoLCA enhance expression of cathelicidin in the airway epithelium via the TGR5-ERK1/2 pathway

**Figure 5:**
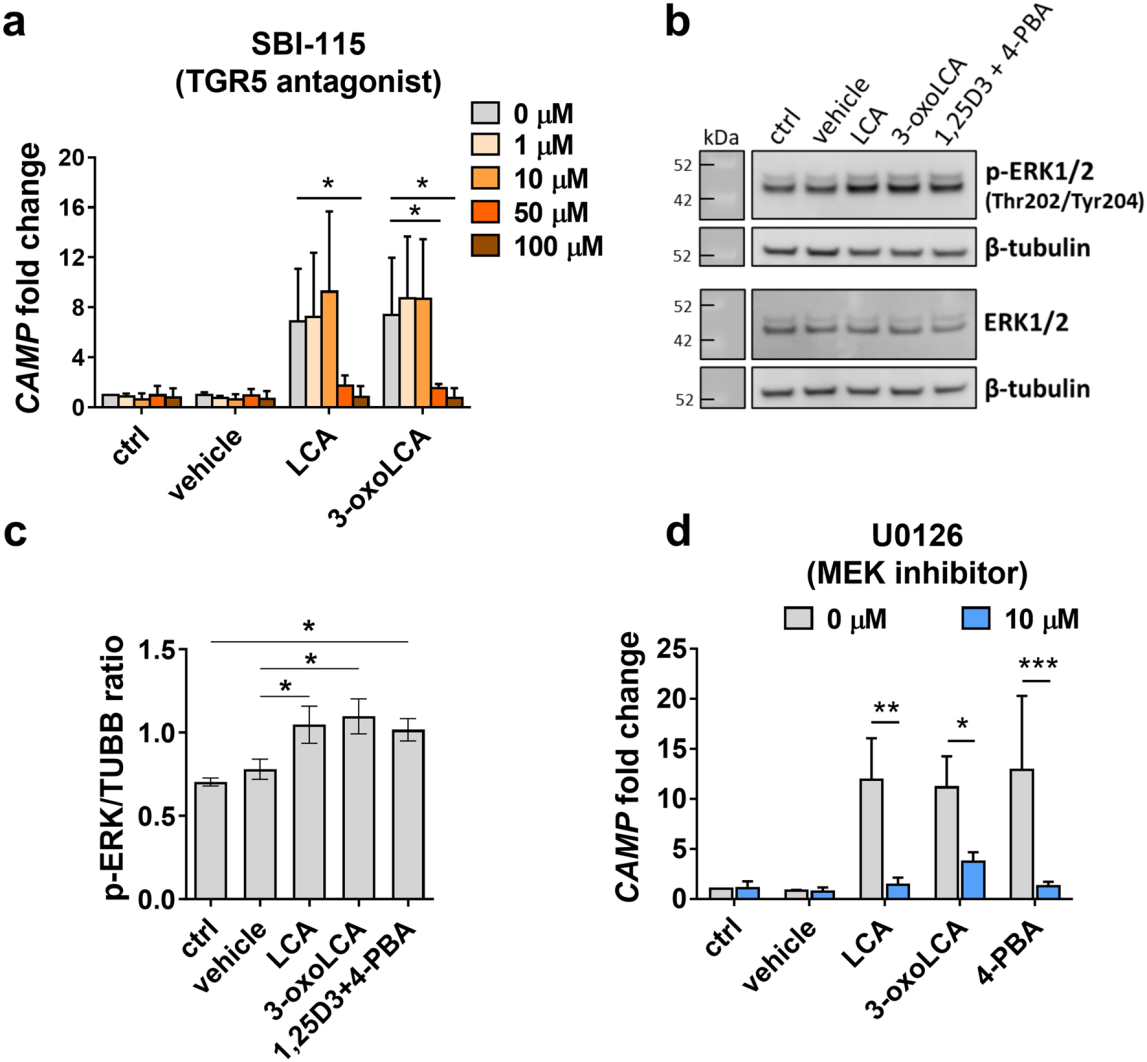
Induction of cathelicidin expression by bile acid metabolites in bronchial epithelium is mediated through the stimulation of TGR5 receptor and activation of the MAPK signaling cascade. (**a**) Expression of cathelicidin (*CAMP*) gene in bronchial epithelial BCi cells pretreated for 1 h with different concentrations of the TGR5 antagonist SBI-115 (1–100 µM) followed by the treatment with lithocholic acid (LCA; 5 µM) and 3-oxolithocholic acid (3-oxoLCA; 5 µM) in the presence of SBI-115 for the next 24 h. (**b**) Analysis of ERK1/2 phosphorylation at Thr202/Tyr204 in comparison to total ERK1/2. Samples for analyses of p-ERK1/2 and ERK1/2 were run on separate gels processed in parallel. After image acquisition the same membranes were washed and incubated with antibodies targeting β-tubulin used as a loading control for each membrane, n = 3 independent experiments. Original blots are presented in Supplementary Fig. 1. (**c**) Quantification of the band intensity presented as a ratio of phosphorylated ERK1/2 at Thr202/Tyr204 to β-tubulin (an internal sample loading control) from n = 3 independent experiments ± SEM analyzed by one-way Anova with Sidak’s post-hoc test, where p values *< 0.05. (**d**) Expression of cathelicidin (*CAMP*) gene in bronchial epithelial cells BCi pretreated for 1 h with MEK inhibitor U0126 (10 µM) followed by treatment with LCA (5 µM) and 3-oxoLCA (5 µM) in the presence of U0126 for the next 24 h. Expression of *CAMP* for all experiments was analyzed by qRT-PCR using *EEF2* housekeeping gene and is presented as a fold change over control (untreated cells) with value 1. All experiments were performed n = 3 independent times ± SD and data were analyzed by two-way Anova with Dunnett’s (SBI-115) and Sidak’s (U0126) post-hoc tests, where* p* values *< 0.05, **< 0.01, ***< 0.001.

After excluding the role of selected nuclear receptors in LCA- and 3-oxoLCA-mediated induction of cathelicidin expression, we investigated the surface G protein-coupled bile acid receptor 1 (TGR5) as a possible target for LCA and 3-oxoLCA in the bronchial epithelium (Fig. 5). The preincubation of BCi cells with different doses of the TGR antagonist SBI-115 blocked the induction of *CAMP* expression by LCA and 3-oxoLCA when SB-115 was used at least at 50 µM (Fig. 5a), showing that LCA and 3-oxoLCA induced cathelicidin expression in bronchial epithelium through stimulation of the TGR5 surface receptor. Therefore, we further delineated the signaling pathway activated by stimulation of TGR5 with LCA and 3-oxoLCA, leading to enhanced cathelicidin expression. In the mouse model of mastitis, stimulation of TGR5 with deoxycholic acid increased the cAMP level^25^ that in another study was shown to induce cathelicidin expression through PKA and ERK-MAPK pathway^26^. Therefore, we analyzed if LCA and 3-oxoLCA activate the ERK1/2 (p44/42 MAPK) pathway in bronchial epithelium leading to the increased *CAMP* expression. Upon treatment with LCA and 3-oxoLCA, the phosphorylation of ERK1/2 at Thr202/Tyr204 residues was increased (Fig. 5b,c), indicating the activation of ERK1/2. This was further confirmed by the ERK1/2 inhibitor, U0126 (MEK inhibitor), that counteracted induction of *CAMP* expression by LCA and 3-oxoLCA (Fig. 5d). Together our data indicate that LCA and 3-oxoLCA induce expression of HDP cathelicidin in bronchial epithelium through the activation of the TGR5-ERK1/2 pathway.

## Discussion

Characterization of microbiota metabolites as signaling molecules affecting host antimicrobial responses are defined only for a handful number of microbial metabolites. For instance, butyrate facilitates the antimicrobial reprogramming of macrophages^27^ and induced expression of cathelicidin antimicrobial peptide in human lung cells^15^. A variety of different microbiota bile acid metabolites circulate in blood^8^ and the knowledge about their biological function related to the modulation of host epithelial immune responses in the GI-distant tissues is scarce. We addressed this question in our study by using lung epithelial cells. We concentrated on modulation of epithelial innate immune responses in respect to the expression of HDPs and used cathelicidin as an example of HDP. Interestingly, LCA and metabolites 3-oxoLCA, AILCA and ILCA induced cathelicidin expression in bronchial epithelium at lower concentrations (Fig. 2) in comparison to the colonic epithelium (Fig. 1). This can be explained by the fact that the primary environment for bile acids and their metabolites is the GI tract, where bile acid metabolites reach approximately 10–50 µM in the colon^1^. In reference to the physiological concentration of bile acid metabolites in the colon, we included 50 µM and higher concentrations that were reported in the literature^28^. In our study, the concentration of 300 and 600 µM inhibited HT-29 cell proliferation and their cytotoxic effects explained the low signal in the CampLuc assay (Fig. 1a). Regarding experiments with human bronchial epithelium, we used higher concentrations of LCA and metabolites (2–5 µM) than those measured in human blood that reached up to 100 nM^8^ to demonstrate the importance of microbial metabolites for modulation of host immune responses^29^ using in vitro models. The differences between physiological concentrations of microbiota metabolites measured in blood and those used for in vitro studies with the lung model also apply to butyrate used at 2–4 mM to induce cathelicidin expression^15,30^, while concentration of butyrate in blood may reach up to 64 µM^31^. The discrepancies between studies with different techniques to measure the concentration of bile acid metabolites in human blood together with great variations in concentrations between individuals^8^ brings the question, what is the actual range of physiological concentrations of bile acid metabolites in human blood. It may be suggested that for example, different diet, microbiota composition, age, metabolic diseases, medication or the “leaky” intestinal barrier will affect concentrations of bile acid metabolites in blood highlighting a possible broad range of physiological concentrations. Interestingly, we found some differences in *CAMP* induction by LCA derivatives between the colonic and lung epithelial cell models. AILCA induced *CAMP* expression in the colon but not in the lung cells and the opposite effect was observed for ILCA. This may be explained by the fact that various microbial metabolites exert different effects depending on the epithelial cell type and a high redundancy of the bile acid receptors^17^. Furthermore, the enhanced expression of the cathelicidin proform at the protein level was confirmed for LCA and 3-oxoLCA in BCi and human primary bronchial/tracheal cells (HBEpC) (Fig. 3). Although the induction of cathelicidin with LCA and 3-oxoLCA was lower than with vitamin D3, it highlighted the physiological relevance of HDP-mediated induction by bile acid metabolites in the airway epithelium.

We were next interested in the mechanism of cathelicidin induction by LCA and 3-oxoLCA in bronchial epithelium. The aryl hydrocarbon receptor (AhR) is a nuclear receptor that works as an environmental sensor of different microbiota metabolites and xenobiotics^18^, therefore we included the AhR antagonist CH223191 in our study. Interestingly, LCA and 3-oxoLCA induced expression of *CYP1B1* gene, one of many AhR downstream target genes; however, induction of cathelicidin by LCA and 3-oxoLCA was not mediated through the AhR (Fig. 4a,b). Several nuclear receptors have been documented targets for different bile acids, especially FXR expressed within the GI-tract^2^ and HT-29 colonic epithelial cell line (Fig. 4c). We did not detect any *FXR* transcript in human primary airway epithelial cells from two different donors and a very low expression level was detected in human bronchial epithelial cell lines BCi and VA10 (Fig. 4c). This was in line with information provided by Human Protein Atlas (www.proteinatlas.org/ENSG00000012504-NR1H4/tissue/lung), showing that FXR is mainly expressed in alveolar cells. The role of FXR in alveolar cells was supported by a study suggesting that inhibition of FXR-mediated signaling might protect from SARS-CoV-2 infection through downregulation of angiotensin-converting enzyme 2 (ACE2) expression in the lung^32^. The low FXR expression in bronchial epithelium indicates that enhanced cathelicidin expression is mediated through the stimulation of a different receptor/-s by bile acids. Both, LCA and 3-oxoLCA were shown to stimulate VDR, that is known to regulate cathelicidin expression in humans^17,24^. However, the stimulation of VDR by bile acid metabolites does not occur in all cell types as showed in the study by Hang et al.^5^, where differentiation of specific subtypes of T cells in response to 3-oxoLCA and AILCA was not mediated through VDR and FXR. In the co-treatment of LCA and 3-oxoLCA with 1,25D3 and 4-PBA, we found an additive effect of LCA and 3-oxoLCA in combination with 1,25D3 on cathelicidin induction at the protein level, in contrast to the synergistic cooperation of 1,25D3 combined with 4-PBA (Fig. 4d,e). In comparison to the treatment with 1,25D3, the addition of LCA and 3-oxoLCA to 1,25D3 did not show significant increase, which may be caused by the fact that 1,25D3 seems to be a stronger inducer of cathelicidin expression than LCA and 3-oxoLCA (Fig. 4d,e). These results would not exclude the VDR as a target receptor for LCA- and 3-oxoLCA-mediated induction of cathelicidin expression in bronchial epithelium. To definitely exclude VDR, we aimed to knockdown the expression of VDR receptor in BCi cells however, satisfactory reduction of the VDR protein level after transfection with several different commercially available siRNA sequences was not reached. Therefore, we investigated stimulation of the possible cell surface receptors^17^. We confirmed that LCA and 3-oxoLCA induce cathelicidin expression through the stimulation of TGR5 receptor in bronchial epithelium (Fig. 5a), what supported our previous findings. The stimulation of TGR5 has been shown to regulate the epithelial barrier function in the gut and recently indicated to have a protective effect against *Staphylococcus aureus*-induced mastitis^25,33^. Since the stimulation of TGR5 increases the cyclic adenosine monophosphate (cAMP) levels shown to regulate *CAMP* expression through PKA and ERK-MAPK pathways^26^, we looked at the activation of the MAPK cascade. Analysis of the phosphorylation level of ERK1/2 kinase (Fig. 5b,c) and the use of MEK inhibitor (Fig. 5d) revealed that LCA and 3-oxoLCA induce expression of HDP cathelicidin in bronchial epithelium through the activation of TGR5-ERK1/2 pathway.

**Figure 6:**
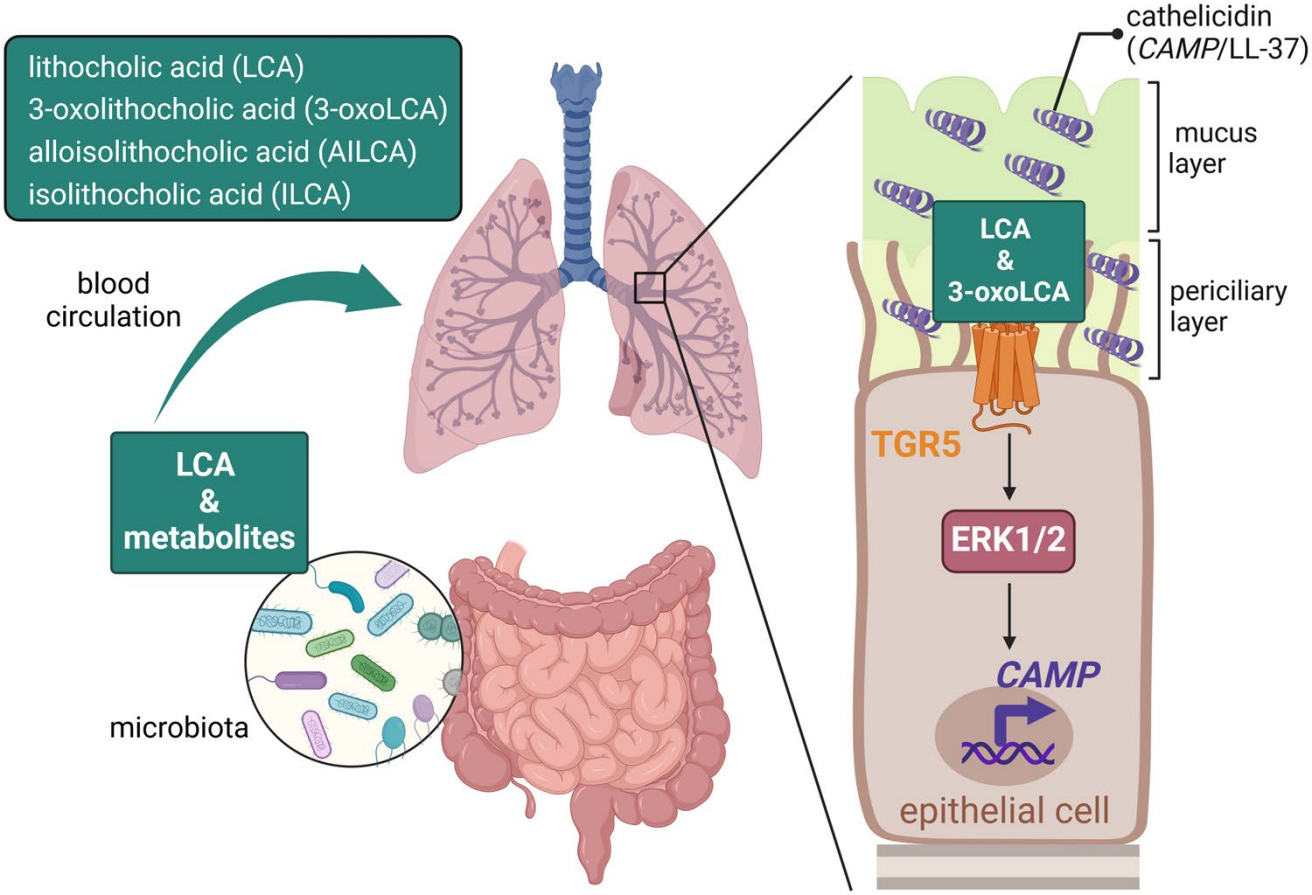
Bile acid metabolites modulate cathelicidin expression in the airway epithelium. Bile acid metabolites, such as lithocholic acid (LCA) and derivatives 3-oxolithocholic acid (3-oxoLCA), alloisolithocholic acid (AILCA) and isolithocholic acid (ILCA) produced by gut microbiota, can enter the bloodstream and low concentrations can be distributed to different organs. In lung, LCA and 3-oxoLCA can induce expression of cathelicidin antimicrobial peptide in airway epithelium through stimulation of the TGR5 surface receptor and activation of the ERK1/2 signaling cascade. Created with www.BioRender.com.

Our study confirmed that bile acid metabolites, produced by gut microbiota entering the blood circulation^8^, act as modulators of antimicrobial response in human airway epithelium, revealing a novel pathway TGR5-ERK1/2 regulating cathelicidin expression (Fig. 6). Although we used only in vitro models, our work serves as a proof-of-concept study characterizing novel mechanisms regulating the frontline epithelial responses in lungs by bile acid metabolites warranting future studies on the gut-lung axis using more physiological models. The importance of our work highlights possible implications of using FDA-approved drugs targeting the Raf/MEK/ERK pathway for cancer therapy^34^. One can speculate that using MEK inhibitors for cancer treatment could possibly decrease expression of antimicrobial effectors in lungs increasing the risk of respiratory infections in cancer patients. Overall, our study highlights bile acid metabolites as modulators of innate immune responses in different cell types through stimulation of different receptors and signaling pathways.

## Methods

### Reagents

All bile acid metabolites were purchased from Cayman Chem. (Cat. No. 20253; 29545; 29542; 29544), 1,25-dihydroxyvitamin D3 from Sigma (D1530) and 4-PBA (Cat. No. 2682) and CH223191 (Cat. No. 3858) from Tocris. The antagonist SBI-115 was purchased from Abcam (ab287087) and MEK inhibitor U0126 from Promega (Cat. No. V112A). Solvents, DMSO and ethanol, were purchased from Santa Cruz (Cat. No. sc-358801) and Sigma (Cat. No. 32221), respectively. Trifluoroacetic acid (TFA; #73645), acetonitrile (#34881) and methanol (#34885) were from Sigma. Fetal bovine serum (Cat. No. 10270106; heat inactivated at 56 °C for 30 min and filtered through 0.45 µm sterile filters) and PBS tablets (Cat. No. 18912014) were from Life Tech. To prepare 1 × Tris-Buffered Saline buffer (TBS), TBS tablets (Merck, Cat. No. 524750-1EA) were dissolved as recommended and Tween 20 (Sigma, Cat. No. P1379) was added to obtain 0.1% final concentration (TBS-T buffer).

### Cell culture

The stable cell line MN8CampLuc-HT-29 was cultured as described previously^14^ except addition of antibiotics and 10% FBS instead of FCS was used. The parental HT-29 cell line (ATCC: HTB-38, batch 70019050) was cultured in McCoy’s 5A (modified) medium (Life Tech., Cat. No. 26600-023) supplemented with 10% FBS (Life Tech., Cat. No. 10270-106), otherwise indicated. Human bronchial/tracheal epithelial primary cells (HBEpC) from two different donors labeled #2644 and #1865 were provided from Cell Applications (Cat. No. 502-05a). BCi-NS1.1^35^, VA10^36^ and HBEpC cells were cultured in Bronchial/Tracheal Epithelial cell growth medium (BEGM) supplemented with retinoic acid (Cell Applications, 511A-500 and 511-RA). HBEpC were sub-cultured using subculture reagent kit (Cell Applications, 090K) and following instructions provided by the vendor. Experiments with SBI-115 antagonist were performed by culturing BCi cells in BEGM BulletKit from Lonza (Cat. No. CC-3170), as BEGM from Cell Applications was temporarily not available from the vendor. Cells were cultured at 37 °C and 5% CO_2_.

### CampLuc assay

The total of 5 × 10^4^ MN8CampLuc-HT-29 cells were seeded on the white flat bottom 96-well plate (Costar, Cat. No. 3917) and 2 days post seeding, cells were incubated with bile acid metabolites for 24 h. Then media were removed, cells were washed with 1 × PBS and lysed in 20 µl of luciferase cell culture lysis reagent for 5 min with gentle agitation on the shaker followed by addition of 100 µl luciferase assay substrate (Promega, Cat. No. E1501) and mixed. The chemiluminescence was measured by Modulus II microplate multimode reader (Turner Biosystems) and chemiluminescent signal was presented as relative luciferase units (RLU).

### Cytotoxicity and proliferation assays

For all cytotoxicity experiments the treatment time was 24 h. All experiments were performed by using CytoTox 96 Non-Radioactive Cytotoxicity assay (Promega, G1780) allowing to measure release of lactate dehydrogenase (LDH) from cells, according to the manufacturer’s instructions with the following modifications. The parental HT-29 cell line was cultured in RPMI 1640 medium (Life Tech., Cat No. 52400041) with 10% FBS. For the treatment with bile acids, RPMI 1640 medium without phenol red (Life Tech., Cat. No. 11835063) supplemented with 5% FBS was used and incubation time was reduced to 15 min to minimize the background signal. The positive LDH control was obtained by freezing the cells with media at − 80 °C. After thawing, media were collected and used as a positive control (100%). Proliferation assays were performed by seeding 2.5 × 10^4^ of BCi and 1 × 10^4^ of HT-29 cells on the 96-well white clear bottom plates (Corning, Cat. No. 3610). The next day (for BCi) or two days (for HT-29) post seeding, cells were treated with bile acid metabolites in the total volume of 100 µl medium for 24 h. The cell proliferation reagent WST-1 (Roche, Cat. No. 05015944001) of 10 µl was added and incubated 30 min at 37 °C and 5% CO_2_. The absorbance was measured at 450 and 620 nm. The proliferation was calculated by subtracting A620 nm from A450 nm values and presented as a percentage of proliferation of control/untreated cells (100%).

### RNA isolation and cDNA preparation

RNA was isolated from approx. 2 × 10^5^ BCi cells by RNeasy Mini Kit (Qiagen, Cat. No. 74104) followed by digestion with 10 units of DNase I (NEB, Cat. M0303L) and RNA cleanup with RNeasy MinElute Cleanup Kit (Qiagen, Cat. No. 74204). The total of 1 µg RNA was used for cDNA synthesis using the High-Capacity cDNA Reverse Transcription Kit (Applied Biosystems, Cat. No. 4368814).

### qRT-PCR

The reaction mix was prepared in the total reaction volume of 10 µl by using PowerUp SYBR Green Master Mix (Applied Biosystems, Cat. No. A25776), 1 µl of cDNA (approx. 50 ng) and primers (Integrated DNA Technologies) at 0.5 µM final concentration. The following primer pairs were used: *CAMP*-for-5ʹ-GCACACTGTCTCCTTCACTG-3ʹ, *CAMP*-rev-5ʹ-CTAACCTCTACCGCCTCCT-3ʹ, *CYP1B1*-for-5ʹ-GAACGTACCGGCCACTATCA-3ʹ, *CYP1B1*-rev-5ʹ-TGGTCACCCATACAAGGCAG-3ʹ, *FXR*-for-5ʹ-ACTGACCTGTGAGGGGTGTA-3ʹ, *FXR*-rev-5ʹ-TGCCCCCGTTTTTACACTTG-3ʹ and *EEF2*-for-5ʹ-GACATCACCAAGGGTGTGCAG-3ʹ, *EEF2*-rev-5ʹ-GCGGTCAGCACACTGGCATA-3ʹ used for the housekeeping gene. The reactions were run in the 7500 Real Time PCR System (Applied Biosystems), using the following cycling conditions: UDG activation at 50 °C for 2 min, polymerase activation at 95 °C for 10 min, 40 cycles of: denaturation at 95 °C for 15 s and annealing at 60 °C for 1 min. The melt curve stage was included (95 °C for 15 s, 60 °C for 1 min, 95 °C for 30 s, 60 °C for 15 s, continuously). The gene expression was calculated using Livak method^37^ and presented as a fold change over control (untreated) cells with value 1. The FXR expression in different cells was calculated using the following formula 2^−ΔCt^ and presented as a relative expression.

### Western blot

For LL-37 Western blot, cell culture media were acidified by addition of 1/1000 volume of 0.1% TFA (v/v in H_2_O) and protein enriched using Oasis HLB 1 cc Vac cartridges (30 mg sorbent per cartridge, 30 µm; Waters, Cat. No. WAT094225). First, columns were activated with acetonitrile, washed three times with 0.1% TFA, acidified samples were loaded on the columns and washed with 0.1% TFA, followed by salt elution with 20% acetonitrile in 0.1% TFA and protein elution with 80% acetonitrile in 0.1% TFA. The eluted fractions were snap-frozen in liquid nitrogen and freeze-dried using Savant Explorer SpeedVac Concentrator (Thermo Fisher). Cells were lysed in RIPA buffer (CST#9806) with Halt Protease Inhibitor Cocktail (Thermo Fisher, Cat. No. 1861278) and Phosphatase Inhibitor Cocktail (CST#5870). The protein enriched pellets reconstituted in water and the cell lysates of 10–20 µg total protein were separated on the 4–12% NuPAGE gradient gels (Life Tech.) as described previously^38^. The primary antibodies, mouse LL-37^39^ (1/1000, 5% milk in TBST-T), rabbit GAPDH (CST#2118, 1/1000, 5% BSA in TBS-T), rabbit phospho-ERK1/2 (CST#4370, 1/2000, 5% BSA in TBS-T), mouse ERK1/2 (CST#4696, 1/2000, 5% milk in TBS-T), rabbit β-tubulin (CST#2128, 1/1000, 5% BSA in TBS-T) were used for overnight incubation at 4 °C with agitation. Membranes were washed four times with TBST-T buffer and incubated at least 1 h at RT with the secondary goat anti-rabbit (Sigma, Cat. No. A0545) or goat anti-mouse (Millipore, Cat. No. AP181P) antibodies conjugated with horseradish peroxidase (HRP) using 1/10,000 dilution in 5% BSA and skimmed milk in TBS-T, respectively. The Western Blotting Luminol Reagent (Santa Cruz, sc-2048) was applied on the washed membranes and the acquisition of the chemiluminescent signal was performed by using ImageQuant LAS 4000 system (G&E Healthcare). The images of the membranes were cropped using GIMP 2.10.32 software and quantification of the band intensity was performed with ImageJ (Fiji) software.

### Statistical analysis and software

The average values from at least three or more independent experiments are shown ± SD. The explicit explanation of the statistical analyses performed using the GraphPad Prism Software version 6 is included in each figure legend text together with *p* values. Western blots were repeated at least three times. Chemical structures were created using ChemSketch, freeware version, Advanced Chemistry Development, Inc. (ACD/Labs), Toronto, ON, Canada, www.acdlabs.com. The scheme in Fig. 6 was created with BioRender.com.

### Supplementary Information

Supplementary Figure 1.

## Data Availability

The data generated and analyzed during the current study are available from the corresponding author on reasonable request.
