# Supplementary material for: Bile acid metabolites enhance expression of cathelicidin antimicrobial peptide in airway epithelium through activation of the TGR5-ERK1/2 pathway
Source: Sci Rep. 2024 Mar 21;14:6750. doi: 10.1038/s41598-024-57251-3 (PMC10957955; doi:10.1038/s41598-024-57251-3)
Supplement: Supplementary file 1 — Supplementary Figure 1. [file 41598_2024_57251_MOESM1_ESM.pdf]

Supplementary Information

Bile acid metabolites enhance expression of cathelicidin antimicrobial peptide in airway epithelium through activation of the TGR5-ERK1/2 pathway.

Iwona T. Myszor[1], Kornelia Lapka[1], Kristjan Hermannsson[1], Rokeya Sultana Rekha[2,3], Peter Bergman[2,3], Gudmundur Hrafn Gudmundsson[1,2,*]

[1] Faculty of Life and Environmental Sciences, Biomedical Center, University of Iceland, Reykjavik, Iceland

[2] Department of Laboratory Medicine, Karolinska Institutet, Stockholm, Sweden

[3] Department of Clinical Immunology and Transfusion Medicine, Karolinska University Hospital, Stockholm, Sweden

[*] Corresponding author: ghrafn@hi.is

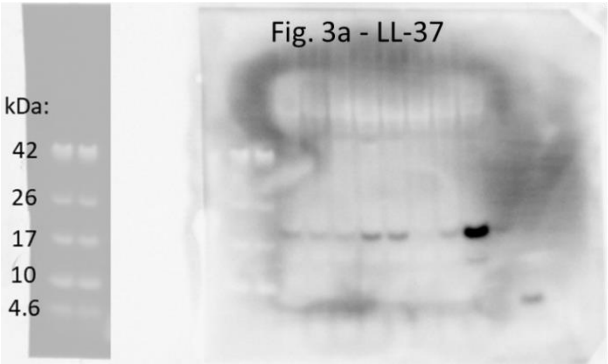
Fig. 3a - LL-37
kDa:
42
26
17
10
4.6

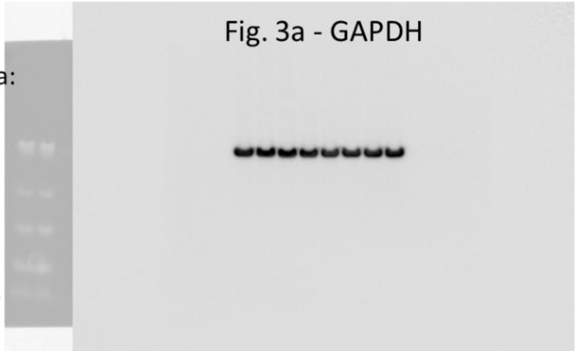
Fig. 3a - GAPDH
kDa:
42
26
17
10
4.6

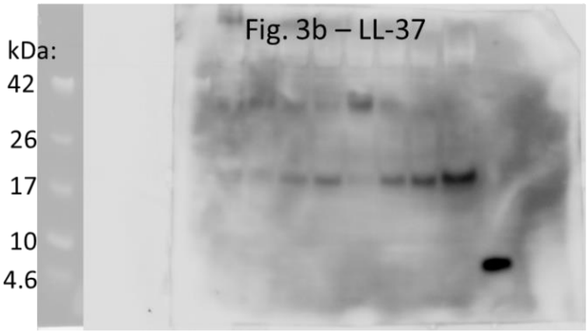
Fig. 3b – LL-37
kDa:
42
26
17
10
4.6

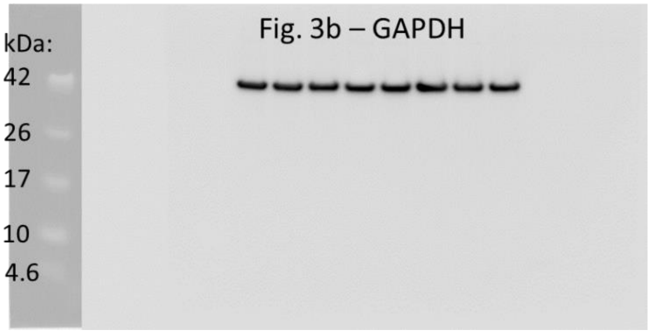
Fig. 3b – GAPDH
kDa:
42
26
17
10
4.6

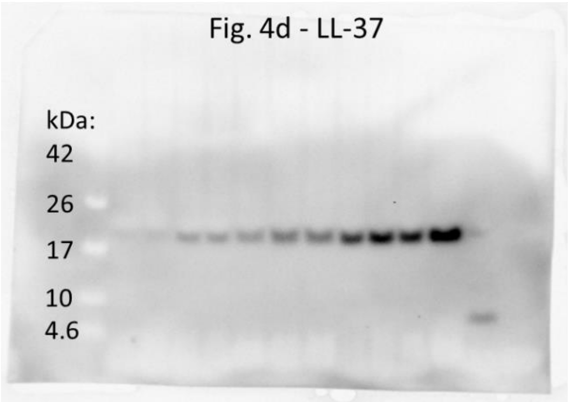
Fig. 4d - LL-37
kDa:
42
26
17
10
4.6

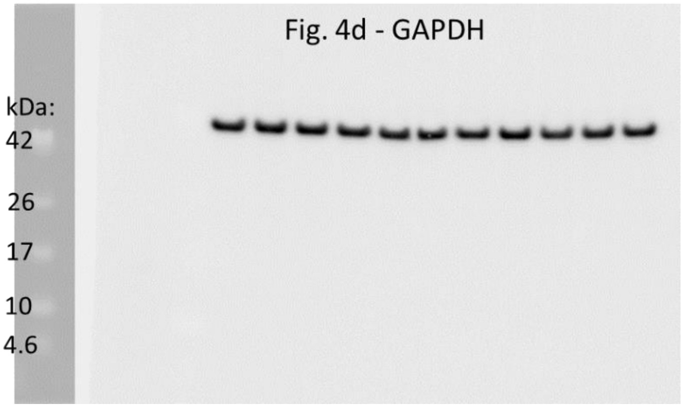
Fig. 4d - GAPDH
kDa:
42
26
17
10
4.6

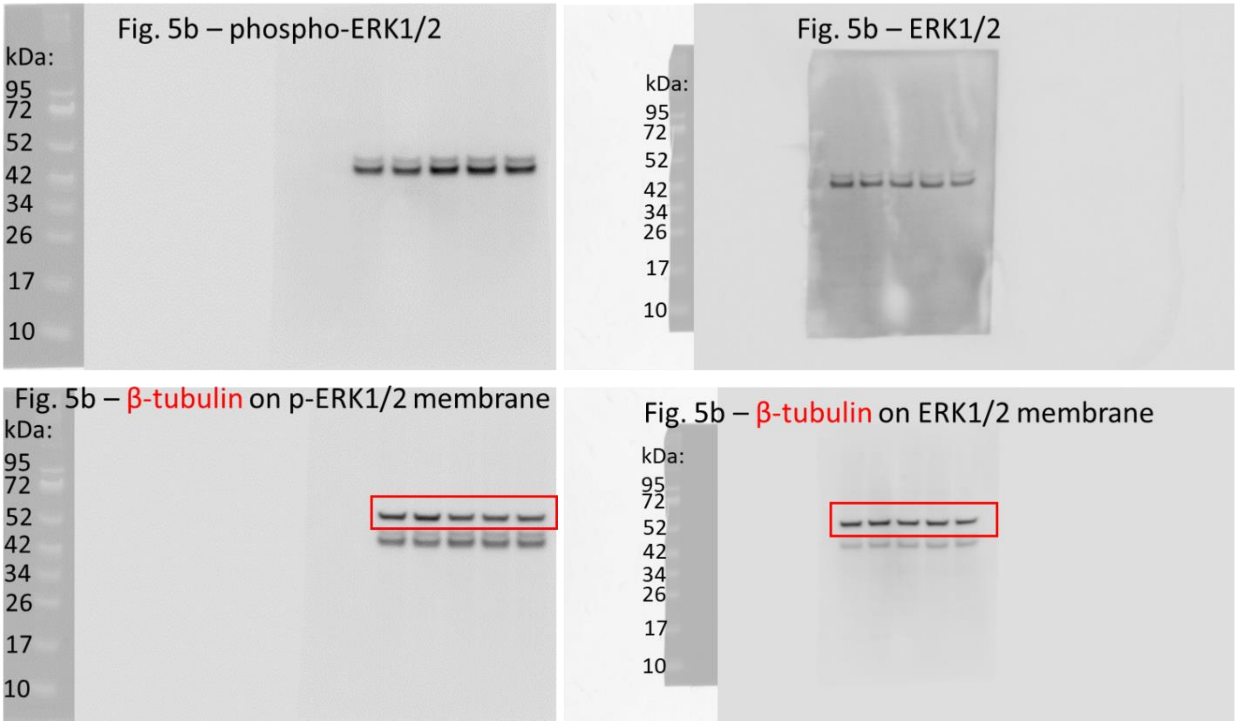

Supplementary Figure 1. The display of full-length Western blots presented in the article.
